# NFAT5 expression in bone marrow-derived cells enhances atherosclerosis and drives macrophage migration

**DOI:** 10.3389/fphys.2012.00313

**Published:** 2012-08-03

**Authors:** Julia A. Halterman, H. Moo Kwon, Norbert Leitinger, Brian R. Wamhoff

**Affiliations:** ^1^Department of Pharmacology, University of Virginia, CharlottesvilleVA, USA; ^2^The Robert M. Berne Cardiovascular Research Center, University of Virginia, CharlottesvilleVA, USA; ^3^Department of Medicine, University of Maryland School of Medicine, BaltimoreMD, USA; ^4^Ulsan National Institute of Science and TechnologyUlsan, South Korea; ^5^Department of Medicine, Cardiovascular Division, University of Virginia, CharlottesvilleVA, USA

**Keywords:** TonEBP, macrophage, atherosclerosis, bone marrow, migration

## Abstract

**Objective**: We have previously shown that the transcription factor, nuclear factor of activated T-cells 5 (NFAT5), regulates vascular smooth muscle cell phenotypic modulation, but the role of NFAT5 in atherosclerosis is unknown. Our main objective was to determine if NFAT5 expression in bone marrow (BM)-derived cells altered atherosclerotic development and macrophage function. **Methods and Results**: NFAT5^+/−^ApoE^−/−^ mice were generated for *in vivo* atherosclerosis studies. Following high fat diet feeding, *en face* analysis of the thoracic aorta established that genome-wide NFAT5 haploinsufficiency reduced atherosclerotic lesion formation by 73%. BM transplant studies revealed that transplantation of NFAT5^+/−^ApoE^−/−^ marrow into NFAT5^+/+^ApoE^−/−^ mice resulted in a similar 86% reduction in lesion formation. *In vitro* functional analysis of BM-derived macrophages demonstrated that NFAT5 is required for macrophage migration, which is a key event in the propagation of atherosclerosis. **Conclusion**: We have identified NFAT5 in BM-derived cells as a positive regulator of atherosclerotic lesion formation and macrophage function in the vasculature.

## Introduction

Nuclear factor of activated T-cells 5 (NFAT5/tonicity-responsive enhancer binding protein) is a Rel homology transcription factor that is critical for the regulation of various cellular functions in both hypertonic and isotonic environments (Miyakawa et al., 1999; Woo et al., 2002; Halterman et al., 2011a). NFAT5 is traditionally known for its hypertonicity-induced activation in tissues that experience large fluxuations in tonicity, such as the kidney (Miyakawa et al., 1999), where hypertonic conditions enhance NFAT5 expression, transactivation, and binding to target genes that control cellular homeostasis (Handler and Kwon, 2001). In tissues that do not undergo large fluctuations in tonicity such as the vasculature, rheumatoid arthritic joints, and carcinomas, various tonicity-independent stimuli have been identified as regulating NFAT5 expression and activity (Jauliac et al., 2002; Yoon et al., 2011; Halterman et al., 2011b). Previous studies in our lab were the first to describe NFAT5 function in the vasculature, and our research has identified a hypertonicity-independent role for NFAT5 in vascular smooth muscle cell (SMC) phenotypic modulation (Halterman et al., 2011b). Additionally, we showed that NFAT5 protein was elevated in ApoE^−/−^ mouse atherosclerotic lesions following a 20-week high fat diet (HFD; Halterman et al., 2011b). However, the role of NFAT5 in chronic atherosclerotic vascular disease is unknown.

Cardiovascular disease is the number one cause of morbidity and mortality worldwide, and atherosclerosis contributes to vascular disease by increasing the risk of vessel occlusion, myocardial infarction, and stroke (Roger et al., 2011). Atherosclerotic lesion formation is propagated by endothelial cell (EC) dysfunction, cholesterol accumulation in the vessel wall, monocyte/macrophage adhesion and migration into the lesion, and SMC formation of a protective fibrotic cap (Lusis, 2000). Although the function of NFAT5 in atherosclerosis is undetermined, NFAT5 regulation in SMCs, ECs, and macrophages has been previously identified outside of the context of vascular disease. Our prior studies show that NFAT5 is required for platelet-derived growth factor BB and serum-induced SMC migration (Halterman et al., 2011b), while others report that NFAT5 knockdown inhibits EC survival and migration, and NFAT5^+/−^ mice have inhibited angiogenesis (Yoon et al., 2011). Research in bone marrow-derived macrophages (BMDMs) highlights the role of hypertonicity-induced NFAT5 activation (Morancho et al., 2008), and NFAT5 protein in macrophages regulates expression of TNFα, VEGF-C, and TLR-induced antimicrobial genes (Machnik et al., 2009; Roth et al., 2010; Buxade et al., 2012).

The main objective of our study was to determine how genome-wide and bone marrow (BM)-specific NFAT5 haploinsufficiency altered atherosclerotic lesion formation. By utilizing NFAT5^+/−^ApoE^−/−^ mice for HFD-induced atherosclerosis studies, we found that genome-wide NFAT5 haploinsufficiency inhibited atherosclerotic lesion development. Furthermore, bone marrow transplant (BMT) of NFAT5^+/−^ApoE^−/−^ BM into NFAT5^+/+^ApoE^−/−^ mice resulted in a similar impairment in lesion development. These results provide evidence that NFAT5 in BM-derived cells is critical for enhancing atherosclerosis. We additionally show that BMDMs harvested from NFAT5^+/−^ mice have impaired migratory abilities, and we believe these findings are noteworthy because macrophage migration into the lesion is a key event in the propagation of atherosclerosis. In summary, we have identified that NFAT5 in BM-derived cells accelerates atherosclerosis, and we highlight the important role of NFAT5 in the regulation of macrophage migration.

## Materials and methods

### Animals

Animal protocols were approved by the University of Virginia Animal Care and Use Committee. NFAT5^+/−^ApoE^−/−^ mice were generated through breeding ApoE^−/−^ (The Jackson Laboratory) and NFAT5^+/−^ mice (gift from H. M. Kwon; Go et al., 2004). *HFD studies*: 22 male littermates were placed on a HFD (by weight 21% fat, 0.15% cholesterol, 19.5% casein, Harlan Labs) for 20 weeks. *BMT studies:* 27 male NFAT5^+/+^ApoE^−/−^ mice underwent irradiation (600 RAD, twice). BM extracted from the femurs and tibias of three male NFAT5^+/+^ApoE^−/−^ donor mice and three male NFAT5^+/−^ApoE^−/−^ donor mice were spun down, resuspended in RPMI media + 5% FBS, and 3 × 10^6^ cells were injected via the tail vein into two groups of recipient NFAT5^+/+^ApoE^−/−^ mice. For proof of radiation efficacy, 2 NFAT5^+/+^ApoE^−/−^ recipients did not receive bone marrow and subsequently died. Following a 4-week recovery, recipient mice were placed on a HFD for 16 weeks.

### En face

Thoracic aortas were fixed in 4% paraformaldehyde, cleaned of all adipose and connective tissue, and stained with Sudan IV for 5 min. The tissues were then longitudinally cut, pinned flat, and imaged. Two different cameras had to be used to image aortas for the first and second *in vivo* studies, which accounts for the slight difference in images observed from one study to the next. Because different cameras were used, we did not cross-analyze quantitative en face data between the two studies. ImageJ software was used for staining quantification.

### Immunohistochemistry

Aortic arches were fixed with 4% paraformaldehyde and embedded in paraffin. Five micrometer sections were prepared for staining with primary antibodies (mouse anti-SMαA [Santa Cruz], mouse anti-MAC2 [Cedarlane]) or hematoxylin and eosin (H&E). Sections cut at the center of the aortic arch (where most lesions develop in the lesser curvature, around 200 um into the tissue) were used for immunohistochemical staining and analysis. In brief, after microwave Antigen Retrieval (Vector Laboratories; with the exception of MAC2), primary antibodies were detected using a Vectastain Elite Kit (Vector Laboratories) and DAB (Dako Corp) for visualization. Harris Hematoxylin (Richard-Allen Scientific) served as a counterstain. Negative controls were run with the omission of the primary antibody.

### Total plasma cholesterol measurement

Blood samples were collected for all mice at the conclusion of HFD feeding. Samples were incubated with the Infinity Cholesterol Reagent (Thermo Scientific) for 5 min at 37°C, and absorbance was measured at 500 nm. All unknown samples were calibrated to a sample of known concentration.

### Quantification of immunohistochemistry and vessel morphometry

Image-Pro Plus 7.0 software was used to quantify SMαA and MAC2 staining in the lesion. ImageJ software was used to determine medial area (=external elastic lamina area-internal elastic lamina [IEL] area) and lesion area (=IEL area-luminal area) from H&E-stained aortic arch sections.

### *In vitro* assays

BM was harvested from the femurs and tibias of 11–13 week-old WT and NFAT5^+/−^ mice. Bones were kept sterile, cut at the ends, and placed into a needle-punctured 500 μl centrifuge tube set within a larger 1500 μl tube. Following high-speed centrifugation, extracted BM cells were treated with 0.83% ammonium chloride to lyse red blood cells. The remaining BM cells were plated onto 100 mm dishes in RPMI Media 1640 (containing 1% antibiotic/antimycotic, 20 mM Hepes, & 10% FBS) plus the addition of macrophage colony stimulating factor (M-CSF, Life, 30 ng/mL). Cells were kept in fresh media containing M-CSF for a period of 7 days to differentiate the cells into BMDMs. *Adhesion assay*: Human umbilical vein endothelial cells (HUVECs) were plated in a 12-well and stimulated with vehicle (diH_2_O, 10 ng/mL) or TNFα (Millipore, 10 ng/mL) in M199 Media for 4 h. BMDMs pre-incubated with Calcein AM (Life, 5 μg) were added to wells containing stimulated HUVECs for a 30-min incubation at 37°. HUVECs were washed thoroughly with PBS and fluorescence was quantified in a FLUOstar plate reader to identify the amount of adherent BMDMs. *Boyden chamber migration assay*: BMDMs in RPMI Media 1640 were plated in the wells of a Costar Transwell (Fisher) and given 24 h to adhere to the membrane. Cells were washed, and RPMI Media 1640 containing M-CSF (Life, 30 ng/mL) or vehicle (diH_2_O, 30 ng/mL) was added to the bottom of the Transwell. Media without M-CSF was added to the top. Cells were incubated for 24 h, washed, and fixed in 4% paraformaldehyde. Cells on the top of the membrane were scraped off with a sterile swab, and migrated cells on the bottom of the membrane were stained with crystal violet, imaged, and quantified using ImageJ software. Control wells were not scraped to verify that BMDMs did originally adhere to the membrane. *BrdU proliferation assay*: BMDMs in RPMI Media 1640 were plated in a 24-well and allowed 24 h to adhere. Cells were washed and RPMI Media 1640 containing M-CSF (Life, 30 ng/mL) or vehicle (diH_2_O, 30 ng/mL) and BrdU (Roche, 10 μm) was added for 24 h. Cells were then washed, fixed in 4% paraformaldehyde, incubated with an anti-BrdU-POD antibody + TMB substrate (Pierce), and absorbance was measured on a FLUOstar plate reader to quantify cellular proliferation. *Engulfment assay:* BMDMs in RPMI Media 1640 were plated in a 24-well and allowed 24 h to adhere. RPMI Media 1640 containing FluoSphere Carboxylate Microspheres (Life, 4.5 × 10^6^ beads per well) was added to wells containing BMDMs for 30 min. BMDMs were thoroughly washed with PBS and fluorescence was quantified to test for engulfment of FluoSpheres. Wells were imaged under a light microscope to verify that no FluoSpheres had simply adhered to the culture dish.

### Statistical analyses

Statistical significance was confirmed through a *t*-test or One-Way ANOVA (*p* < 0.05).

## Results

### Genome-wide NFAT5 haploinsufficiency inhibits atherosclerotic lesion formation

**Figure 1:**
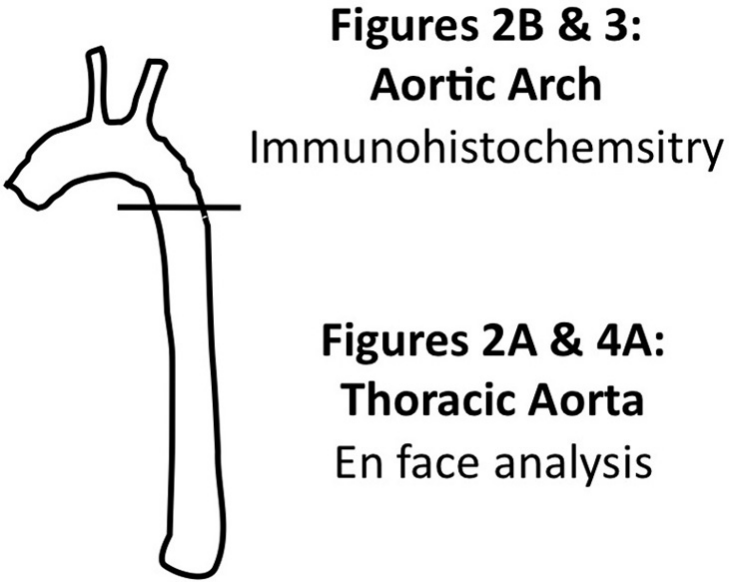
**Schematic of artery preparations.** Mouse aortic tissues were handled identically for both genome-wide and bone marrow transplant atherosclerosis studies. Aortic arches were fixed and sectioned for immunohistochemical analysis. Descending thoracic aortas were fixed, cleaned and stained with Sudan IV for en face analysis.

**Figure 2:**
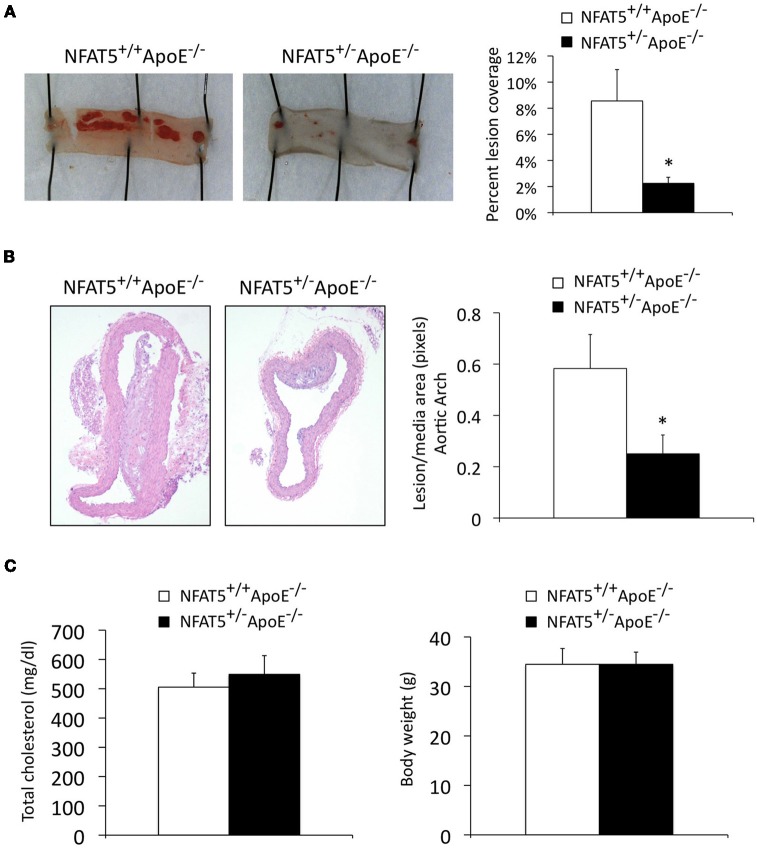
**Genome-wide NFAT5 haploinsufficiency inhibits atherosclerotic lesion formation.** Mice were placed on a high fat diet for 20 weeks. **(A)** En face Sudan IV staining and analysis of the thoracic aorta. **(B)** H&E staining and analysis of aortic arch lesions. **(C)** Total cholesterol and mouse body weight were measured after 20 weeks of high fat diet feeding (*n* = 7 NFAT5^+/+^ApoE^−/−^ mice and 11 NFAT5^+/−^ApoE^−/−^ mice, ^*^p < 0.05).

Due to the perinatal lethality of our NFAT5^−/−^ mice, NFAT5^+/−^ApoE^−/−^ mice were generated for atherosclerosis studies. Aortic arches were harvested for immunohistochemical analysis, and descending thoracic aortas were stained with Sudan IV and laid en face for lesion identification (Figure 1). En face analysis identified that NFAT5^+/−^ApoE^−/−^ thoracic aortas displayed a 73% reduction in atherosclerotic lesion coverage compared to NFAT5^+/+^ApoE^−/−^ controls (Figure 2A). Similarly, H&E staining of lesions in the lesser curvature of the aortic arch identified a 43% reduction in lesion/media area in NFAT5^+/−^ApoE^−/−^ aortas (Figure 2B). Following 20 weeks of HFD feeding, no difference was recorded in total cholesterol or body weight between NFAT5^+/−^ApoE^−/−^ and control NFAT5^+/+^ApoE^−/−^ mice (Figure 2C).

### Immunohistochemical quantification of aortic arch lesions does not reveal a discernable difference in smooth muscle cell or macrophage investment of the lesion

**Figure 3:**
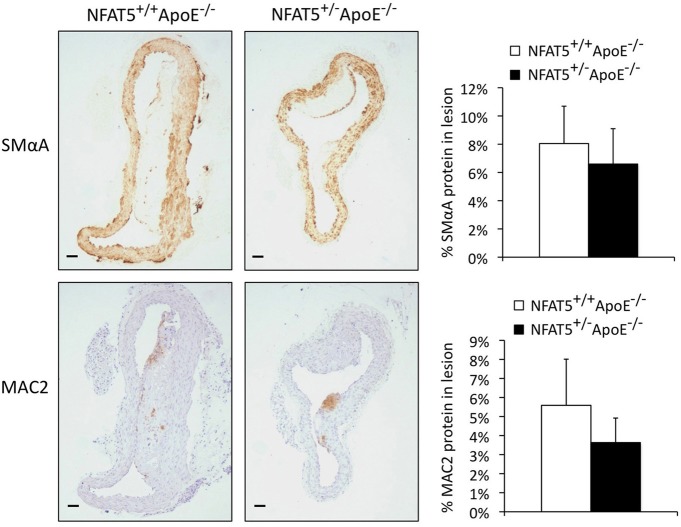
**Immunohistochemical quantification of aortic arch lesions does not reveal a discernable difference in smooth muscle cell or macrophage investment of the lesion.** Immunohistochemical analysis of aortic arch lesions. SMαA and MAC2 are markers for smooth muscle cells and macrophages, respectively (*n* = 7 NFAT5^+/+^ApoE^−/−^ mice and 11 NFAT5^+/−^ApoE^−/−^ mice, bar = 0.6 μm).

To examine whether NFAT5 levels had an effect on lesion composition or cellular content, we performed immunohistochemistry to quantify SMC and macrophage investment of aortic arch lesions (Figure 3). Smooth muscle alpha actin (SMαA) and macrophage 2 (MAC2) protein identified SMCs and macrophages, respectively, that had migrated into the lesion. With this method of analysis, we were unable to record a significant difference in SMC or macrophage investment of the lesion (Figure 3). Further cell-specific NFAT5 haploinsufficient *in vivo* experiments were, therefore, required to determine how NFAT5 deficiency in the cells of the lesion affected atherosclerosis progression.

### Transplantation of NFAT5 haploinsufficient bone marrow inhibits atherosclerotic lesion formation

**Figure 4:**
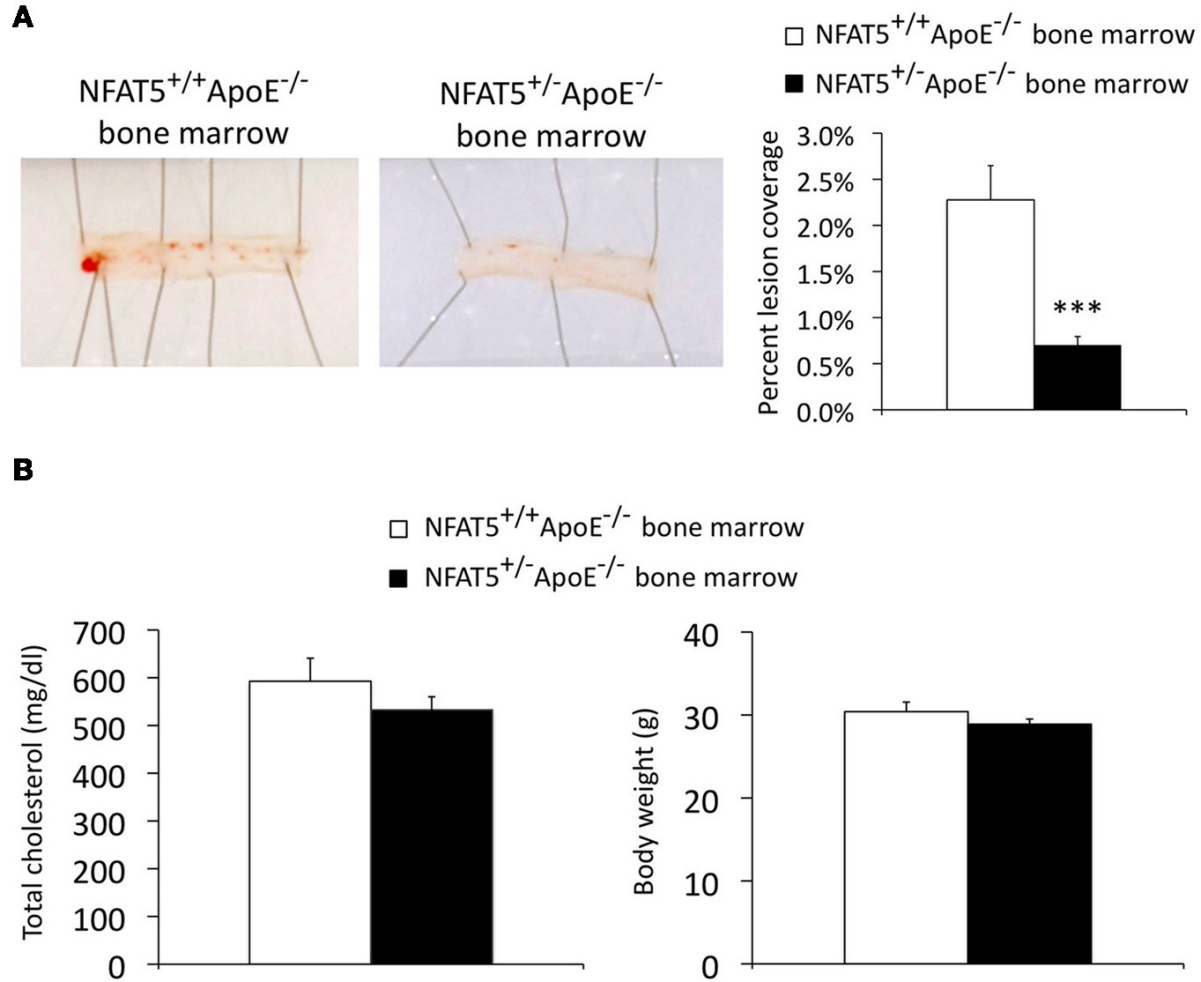
**Transplantation of NFAT5 haploinsufficient bone marrow inhibits atherosclerotic lesion formation.** Bone marrow transplant mice were placed on a high fat diet for 16 weeks. **(A)** En face Sudan IV staining of the thoracic aorta. **(B)** Total cholesterol and mouse body weight were measured after 16 weeks of high fat diet feeding (*n* = 10 NFAT5^+/+^ApoE^−/−^ BM mice and 15 NFAT5^+/−^ApoE^−/−^ BM mice, ^***^p < 0.001).

We sought to establish if NFAT5 levels in BM-derived cells alone could account for the reduction in atherosclerosis observed in NFAT5 haploinsufficient mice. To test this hypothesis, we performed a BMT of NFAT5^+/−^ApoE^−/−^ BM into NFAT5^+/+^ApoE^−/−^ mice. Following a 4-week recovery and 16-week HFD, mouse aortas were prepared for analysis (Figure 1). Sudan IV staining and en face preparation of the thoracic aorta identified an 86% reduction in atherosclerotic lesion coverage in mice transplanted with NFAT5^+/−^ApoE^−/−^ BM (Figure 4A). Unlike our genome-wide NFAT5 haploinsufficient atherosclerosis studies, mice transplanted with NFAT5^+/−^ApoE^−/−^ BM developed little to no lesions in the aortic arch and therefore could not be used for immunohistochemistry-based lesion composition analysis. After 16 weeks of HFD feeding, no difference was recorded in total cholesterol or body weight between both BMT groups (Figure 4B).

### NFAT5 deficiency impairs macrophage migration

**Figure 5:**
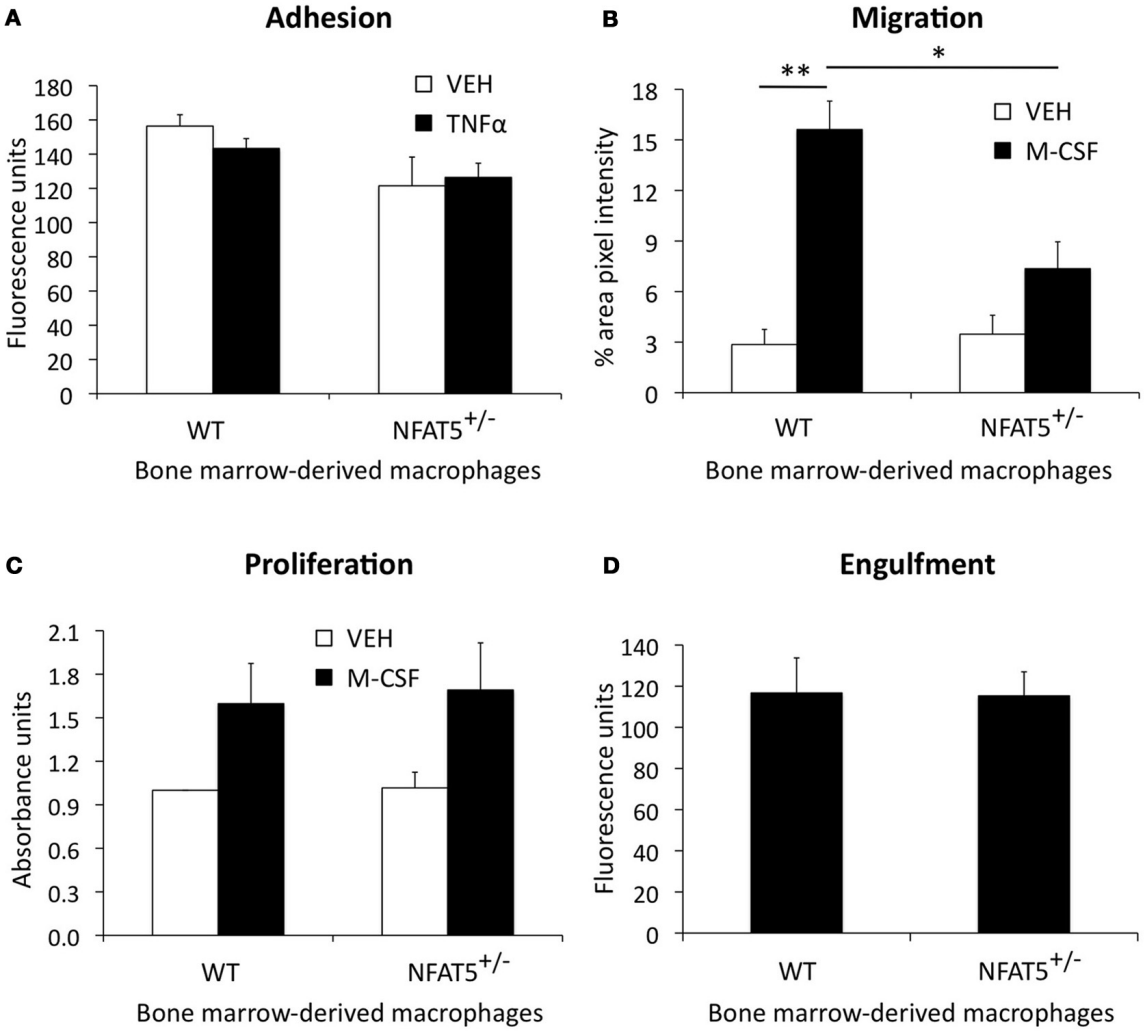
**NFAT5 deficiency impairs macrophage migration.** Bone marrow harvested from WT and NFAT5^+/−^ mice was treated with M-CSF for 7 days to differentiate cells into bone marrow-derived macrophages (BMDMs). Functional analyses were then performed. BMDMs were tested for adhesion to TNFα-activated endothelial cells **(A)**, M-CSF-stimulated chemotactic migration **(B)**, M-CSF-stimulated proliferation **(C)**, and engulfment of fluorescent beads **(D)** (*n* = 3−6 WT or NFAT5^+/−^ mice, ^*^p < 0.05 ^**^p < 0.005).

Results from our BMT studies demonstrate that NFAT5 expression in BM-derived cells is required for atherosclerosis development in the mouse aorta (Figure 4A). Although the BM is comprised of many different cell types (i.e., macrophages, T-cells, B-cells, dendritic cells, mast cells, and neutrophils), these cells have varying degrees of involvement in atherosclerosis (Hansson and Hermansson, 2011). Macrophages play the most dominant role in enhancing lesion size, instability, and vascular inflammation (Moore and Tabas, 2011), which led us to question if NFAT5 expression in macrophages was required for their normal function in atherosclerosis. These functions are: (1) adhesion to ECs in atherosclerosis-prone regions of the vasculature, (2) chemotactic migration toward cytokines produced in the lesion, (3) cytokine-stimulated proliferation, and (4) engulfment of particles. BM harvested from WT and NFAT5^+/−^ mice was differentiated into BMDMs for *in vitro* analysis. Our results show that NFAT5 is not required for macrophage adhesion (Figure 5A), proliferation (Figure 5C), or engulfment (Figure 5D), but NFAT5 is required for macrophage migration (Figure 5B). NFAT5^+/−^ BMDMs exhibited significantly decreased chemotactic migration in response to M-CSF stimulation compared to control WT BMDMs (Figure 5B).

## Discussion

Atherosclerosis is a complex, chronic inflammatory disease. Various BM-derived immune cells such as macrophages, B-and T-cells, and mast cells have been implicated in atherosclerotic development and inflammation (Hansson and Hermansson, 2011). Macrophages constitute the largest fraction of immune cells recruited to the lesion and are responsible for the release of inflammatory cytokines, cholesterol uptake, and lesion necrosis. Our studies reveal that NFAT5 expression in BM-derived cells alone is responsible for enhancing atherosclerosis. While it is known that NFAT5 regulates inflammatory gene expression in macrophages (Machnik et al., 2009; Roth et al., 2010; Buxade et al., 2012), we show novel evidence for the NFAT5-dependent regulation of macrophage function. We report that NFAT5 is required for M-CSF-induced macrophage chemotactic migration, which suggests that NFAT5 may play a role in M-CSF-mediated recruitment of monocytes/macrophages into the atherosclerotic lesion. We hypothesize that NFAT5 haploinsufficiency *in vivo* may result in deficient macrophage migration into the lesion, therefore leading to decreased macrophage uptake of cholesterol, decreased foam cell formation, and an overall reduction in lesion size.

Our lab has previously reported that NFAT5 is required for SMC migration *in vitro* in response to platelet-derived growth factor BB (PDGF-BB) stimulation (Halterman et al., 2011b). PDGF-BB is a mitogen released in the vasculature following acute vascular injury; however, PDGF-BB does not play as central a role in chronic atherosclerosis. We were unable to measure a significant difference in SMC investment of NFAT5^+/+^ApoE^−/−^ and NFAT5^+/−^ApoE^−/−^ lesions through our method of analysis, but generation of a SMC-specific NFAT5^+/−^ApoE^−/−^ mouse would provide a model to specifically determine the role of NFAT5 in SMCs of the atherosclerotic lesion. We chose to proceed with BM transplant studies to specifically analyze how NFAT5 deficiency in BM-derived cells impacted atherosclerotic development.

Although undetermined, NFAT5 regulation of B- and T-cells in atherosclerosis could also account for the drastic reduction in lesion formation identified in NFAT5 haploinsufficient BM transplant mice. Previous studies demonstrate that *in vitro* stimulation of NFAT5^−/−^ T-cells in hypertonic media results in an imbalanced naïve/memory T-cell response (Berga-Bolanos et al., 2010). It is, therefore, possible that mice receiving NFAT5 haploinsufficient BM have decreased T-cell investment of the lesion due to impaired T-cell memory. Further studies will be necessary to determine the precise role of NFAT5 expression in T-cells of the atherosclerotic lesion.

We show that NFAT5 is required for M-CSF-induced macrophage migration, but it is unknown how M-CSF stimulation regulates NFAT5 expression, activation, or cellular localization. NFAT5 expression in macrophages has only been identified as being regulated by toll-like receptor stimulation (Buxade et al., 2012) and hypertonicity-induced activation of NFAT5 (Morancho et al., 2008; Machnik et al., 2009). Although untested, M-CSF and other atherogenic stimuli may be involved in the activation of downstream kinases to promote the phosphorylation and translocation of NFAT5 to the nucleus. This putative mechanism could result in the NFAT5-mediated activation of migratory genes such as Cyr61, a known NFAT5 target gene in skeletal muscle myoblasts (O'Connor et al., 2007). While it would be difficult to test, we speculate that hypertonic microenvironments could develop within the vessel wall during atherosclerotic lesion progression, possibly due to foam cell necrosis and the subsequent release of intracellular contents into the extracellular space. Other studies demonstrate that macrophages in a hypertonic environment increase NFAT5 expression and NFAT5 binding to the vascular endothelial growth factor C (VEGF-C) promoter to enhance lymphogenesis in the skin (Machnik et al., 2009). VEGF-C is also a potent inducer of vascular angiogenesis that stimulates intimal neovascularization (i.e., increased formation of blood vessels within the atherosclerotic lesion) to enhance lesion progression, macrophage apoptosis, and lesion instability (Nakano et al., 2005; Schmeisser et al., 2006). Therefore, small hypertonic microenvironments within atherosclerotic lesions could serve to activate NFAT5 in macrophages, then in turn enhancing VEGF-C expression, angiogenesis, and lesion development.

Together, these data provide novel insight into the NFAT5-dependent regulation of atherosclerotic lesion development and macrophage function. The results of these studies demonstrate the importance of NFAT5 expression in the immune cells associated with chronic vascular disease and provide evidence for the role of NFAT5 in inflammation and atherosclerosis.

### Conflict of interest statement

The authors declare that the research was conducted in the absence of any commercial or financial relationships that could be construed as a potential conflict of interest.
